# Binge drinking during adolescence and young adulthood is associated with deficits in verbal episodic memory

**DOI:** 10.1371/journal.pone.0171393

**Published:** 2017-02-02

**Authors:** Carina Carbia, Fernando Cadaveira, Francisco Caamaño-Isorna, Socorro Rodríguez-Holguín, Montse Corral

**Affiliations:** 1 Department of Clinical Psychology and Psychobiology, Universidade de Santiago de Compostela, Santiago de Compostela, Spain; 2 Consortium for Biomedical Research in Epidemiology & Public Health (CIBERESP), Department of Preventive Medicine, Universidade of Santiago de Compostela, Santiago de Compostela, Spain; Oregon Health and Science University, UNITED STATES

## Abstract

Binge drinking (BD), a harmful pattern of alcohol consumption, is common during adolescence. Young adults with alcohol use disorders exhibit hippocampal alterations and episodic memory deficits. However, it is not known how these difficulties progress in community BD adolescents. Our objective was to analyze the relationship between BD trajectory and verbal episodic memory during the developmental period spanning from adolescence and to early adulthood. An initial sample of 155 male and female first-year university students with no other risk factors were followed over six years. Participants were classified as stable non-BDs, stable BDs and ex-BDs according to the third AUDIT item. At baseline, participants comprised 36 ♂/ 40 ♀ non-BDs (18.58 years), 40 ♂/ 39 ♀ BDs (18.87 years), and at the third follow-up, they comprised 8 ♂/ 8 ♀ stable non-BDs (25.49 years), 2 ♂/ 2 ♀ stable BDs (25.40) and 8 ♂/ 12 ♀ ex-BDs (24.97 years). Episodic memory was assessed four times with the Logical Memory subtest (WMS-III) and the Rey Auditory Verbal Learning Test (RAVLT). Generalized linear mixed models were applied. The results showed that, relative to non-BDs, stable BDs presented difficulties in immediate and delayed recall in the Logical Memory subtest. These difficulties remained stable over time. The short-term ex-BDs continued to display difficulties in immediate and delayed recall in the Logical Memory subtest, but long-term ex-BDs did not. The effects were not influenced by age of alcohol onset, frequency of cannabis use, tobacco use or psychopathological distress. In conclusion, BD during adolescence and young adulthood is associated with episodic memory deficits. Abandoning the BD pattern may lead to partial recovery. These findings are consistent with the vulnerability of the adolescent hippocampus to the neurotoxic effects of alcohol.

## Introduction

Alcohol is the most widely used psychoactive drug among young people and its excessive consumption represents a serious health challenge [1]. Binge drinking (BD) or heavy episodic drinking is recognized as a common pattern of alcohol consumption among university students [2]. This pattern of consumption brings blood alcohol concentration (BAC) levels to 0.8 g/l [3]. BD peaks during late adolescence [4] when significant neuromaturational changes take place [5], including synaptic pruning, myelinization, stronger functional inter-regional connectivity and high hippocampal plasticity [6, 7]. The adolescent brain may therefore be particularly susceptible to the detrimental effects of alcohol [8, 9].

The findings of animal studies indicate that, relative to adult rats, adolescent rats are particularly vulnerable to ethanol neurotoxicity [10, 11]. Most previous studies have primarily focused on two regions traditionally linked to alcoholism-related alterations in adult clinical populations: the prefrontal cortex (PFC) and the hippocampus [12, 13]. Adolescent rodent models of BD have revealed frontal cortical alterations [10, 14, 15]. In humans, structural and functional imaging data of adolescents with Alcohol Use Disorder (AUD) have shown prefrontal abnormalities, both prior to alcohol misuse and as a consequence of heavy drinking [13]. Likewise, studies based on non-clinical samples have shown that adolescent BDs have thicker cortices in left frontal regions [16] and larger volume in the dorsolateral prefrontal cortex [17], which is associated with poorer neuropsychological performance. These findings have been interpreted as an alcohol-related neuromaturational delay; altered typical brain development. Neuropsychological studies have revealed that BDs have poor executive functions (especially in working memory) [18–20], which are typically dependent on the PFC.

Regarding the hippocampus, recent evidence from animal models has revealed long-term reductions in hippocampal neurogenesis [21] and persistent deficits in hippocampal-dependent memory [22, 23]. Hippocampal neurogenesis occurs at a higher rate in adolescents than in adults. Thus, repeated episodes of excessive alcohol intake may damage the hippocampus, leading to cell death and inhibition of neurogenesis [7]. Human studies support the notion that chronic alcoholism is associated with neurodegeneration, reductions in the volume of the hippocampus [12, 24] and deficits in hippocampal-dependent memory, such as episodic memory deficits and verbal learning difficulties, among others [25]. Young adults diagnosed with AUD have a smaller hippocampal volume than healthy individuals [26], and greater differences are observed in the left hemisphere [27, 28]. Accordingly, young people with AUD perform poorly in episodic memory tests [29, 30]. Although verbal memory has classically been associated with correct functioning of the hippocampus, it also depends on frontal-temporal interactions [31]. The medial temporal lobe and the PFC both contribute in different ways to the processes of encoding, storage and retrieval of episodic information [32,33].

Adolescents in the general population engaging in BD also exhibit episodic memory deficits [34] and executive difficulties that contribute to poor verbal memory performance in semantically-related list-learning tasks [35–37]. In two previous studies carried out with part of this sample and using the Rey Auditory Verbal Learning Test (RAVLT) (a list of non-semantically related words), we found that BDs remembered fewer words from list B (short-term attention/memory span) and displayed greater proactive interference [38]. However, these differences disappeared after maintenance of the BD pattern of alcohol consumption for two years [39]. These errors in the RAVLT may be interpreted as executive difficulties rather than episodic memory deficits, as also suggested in several studies [35–37]. The fact that the executive difficulties disappeared after two years (interpreted as an improvement) may suggest an alcohol-related delay in neuromaturation, principally affecting prefrontal regions and resulting in BDs gaining executive efficiency later than age-matched non-BDs.

List-learning tasks such as the RAVLT and story recall tasks such as the Logical memory subtest (WMS-III) are some of the most common verbal memory tasks, but they are not interchangeable as each measures different memory processes. The RAVLT is an unstructured word list that requires implementation of strategies (e.g., semantic organization) for good performance, which is associated with the frontal lobe [40, 41]. The LM is a story-learning task with a structured context that has been shown to be dependent on the functioning of the left medial temporal lobe, which plays a key role in memory consolidation [42, 43]. Numerous studies have reported that frontal patients display impaired performance on list-learning tasks [40, 44–46]. Moreover, patients with executive dysfunctions showed poor performance of list-learning tasks (California Verbal Learning Test (CVLT]), but not of logical memory tasks [47, 48]. In addition, the performance of list-learning tasks by frontal patients has been characterized for increased susceptibility to interference [49] perseverative errors [50] and intrusions [51, 52]. In the RAVLT these type of errors are usually interpreted in terms of impairments in self-monitoring functions [52–54]. Prefrontal or hippocampal–dependent deficits may lead to different difficulties in verbal memory tasks.

Using a story-learning paradigm, we observed that BDs performed poorly in immediate and delayed recall of verbal information in the Logical Memory subtest (WMS-III), which is a classic hippocampal-dependent task proposed as the “purest” measure of episodic memory as it showed the lowest degree of overlap with other non-episodic memory measures [55]. The deficits persisted after two years of BD [38, 39]. These findings are consistent with the vulnerability of the adolescent hippocampus to the neurotoxic effects of alcohol. To the best of our knowledge, no longitudinal studies have yet assessed the impact of BD trajectory in episodic memory in adolescents without other risk factors. In a 10-year follow-up study, the authors reported that verbal memory continued to decline in clinical youths with a chronic trajectory of alcohol and other drug use (AOD) [56]. Nonetheless, the extent to which abandoning the BD pattern of consumption in healthy adolescents enables recovery of memory performance remains unknown.

The aim of the present study was to elucidate the relationship between BD trajectory and verbal episodic memory during the developmental period spanning from adolescence to early adulthood. For this purpose, we analyzed data from a 6-year longitudinal study, following healthy participants between the ages of 18 and 23 years. In addition, we assessed whether the association between BD trajectory and episodic memory is modulated by sex, age of alcohol use onset, psychopathological symptoms or frequency of cannabis use and tobacco use. We thus tested the following hypotheses: (I) in a list-learning paradigm with non-semantically related words (RAVLT), BDs will show difficulties related to prefrontal dysfunction; (II) in a story-learning paradigm (Logical Memory, WMS-III) BDs will show difficulties in verbal episodic memory that are compatible with hippocampal dysfunction; (III) the progression of difficulties related to prefrontal regions will improve over time, whereas difficulties related to medial temporal lobe dysfunctions will deteriorate further or remain stable, suggesting different possible mechanisms underlying neurocognitive damage; and finally, (IV) performance of these tasks will improve in subjects who abandon the BD pattern of consumption, indicating partial recovery.

## Materials and Methods

### Participants

The sample initially comprised 155 healthy university students (76 males and 79 females). To select the sample, an anonymous questionnaire, including the Alcohol Use Disorders Identification Test (AUDIT) [57] and other questions about substance use, was administered to first-year students in class (for further details see [58]. Participants were classified according to their answers to two items: “How often do you have six or more drinks on a single occasion? Never/Less than monthly/Monthly/Weekly/Daily or almost daily” (which is the third item of the AUDIT) and the speed of consumption (drinks per hour). In Spain, a standard alcoholic drink contains 10 g of ethanol. Accordingly, drinking six beverages at a speed of more than two drinks per hour would thus lead to a blood alcohol concentration (BAC) of around 0.8 g/l or higher, considered a BD episode in the present study.

### Procedure

Participants were followed up during a 6-year period (between ages of 18 and 23 years), and four evaluations were made on average every 22 months. As the objective was to assess the effects of the BD trajectory, participants were classified as stable non-BDs (who remained as non-BDs during all follow-ups), stable BDs (who continued as BDs throughout the 6 years) and ex-BDs (who abandoned the BD pattern of alcohol consumption at the first, second or third follow-up). The ex-BDs were further classified into two trajectories: short-term ex-BDs (first time/evaluation with this status) and long-term ex-BDs (more than one evaluation with this status) to examine any changes related to the length of time without BD episodes. The non-BDs were defined as those who never consumed 6 drinks on one occasion (or less than monthly) and who consumed alcohol at a speed of two drinks or less per hour. The BDs consumed 6 drinks on one occasion monthly or weekly and the speed of alcohol consumption was three drinks or more per hour. They were excluded if the AUDIT total score was >20. The ex-BDs consumed 6 drinks on one occasion less than monthly and/or they consumed two drinks or less per hour. Abstainers were not included in the sample.

The number of participants decreased throughout the study: 155 (76 non-BDs/ 79 BDs) at baseline; 93 (39 stable non-BDs/ 33 stable BDs/ 21 ex-BDs) at the first follow-up; 74 (33 stable non-BDs/ 17 stable BDs/ 24 ex-BDs) at the second and 40 (16 stable non-BDs/ 4 stable BDs/ 20 ex-BDs) at the final follow-up. The total number of data points in each trajectory was 164 stable non-BDs; 133 stable-BDs and 65 ex-BDs (grouped as 33 short-term ex-BDs and 32 long-term ex-BDs). The classification criteria did not allow transitions in the trajectories (e.g. a non-BD who changed to BD at the second evaluation would be excluded from the analysis in the following evaluations but maintained for the previous evaluations). Given that this classification implies sample attrition over time, we performed a statistical analysis allowing transitions in consumption trajectory (e.g. a non-BD in the first evaluation who changed to BD in the second assessment was then considered a BD at that specific time point) to ensure that this method of classification did not have any relevant influence on the results. In other words, the statistical model considered the specific pattern of consumption at each time point. The results obtained were almost identical. We used the stable group classification, for the sake of simplicity.

The following exclusion criteria were considered in each of the evaluations to reduce relevant confounding factors: history of neurological disorders; psychiatric disorders (DSM-IV-TR), including attention-deficit hyperactivity disorder, conduct disorder or previous diagnosis of depression or anxiety; current psychopathological symptoms as assessed by the Symptom Checklist-90-R (SCL-90-R) [59] (participants were excluded if they had scores above 90th in the Global Severity Index [GSI] or in at least two symptomatic dimensions); alcohol use disorders; substance use (i.e. opiates, hallucinogens, cocaine, amphetamines or medically prescribed psychoactive substances), except nicotine and cannabis (daily cannabis users were excluded). The item in the questionnaire regarding drug use was “Which of these substances have you consumed and how frequently do you consume them? Hallucinogens, ecstasy, cocaine, heroin, amphetamines, speed, cannabis and tobacco. Other exclusion criteria used were motor or sensory deficits; family history of alcoholism in first-degree relatives or other major psychopathological disorder (depression, anxiety, schizophrenia diagnoses etc.) in first degree relatives.

In each of the four evaluations, a neuropsychological battery was administered together with an interview in which the same exclusionary criteria were considered in order to yield a sample of healthy university students with no other risk factors. Only those participants included in the previous evaluation were contacted again for each new evaluation. This means that participants who underwent the final evaluation had also undergone all previous assessments. The Vocabulary subtest of the WAIS-III [60] was used to estimate the intellectual level of the participants. All participants gave written informed consent and received monetary compensation for their participation. The study was approved by the Bioethics Committee of the University of Santiago de Compostela, according to the principles expressed in the Declaration of Helsinki.

### Materials

The Rey Auditory Verbal Learning Test (RAVLT) [61]. This task consists of remembering 15 semantically unrelated words (List A), which are read out loud in 5 consecutive trials. After each trial the subject is asked to recall as many of the words as possible. An interference list (List B) was then presented and free-recall tested. The subject was then again asked to recall the words on list A without these words having been presented a second time (Trial VI). After 30 minutes, subjects were asked for their free recall of list A (Trial VII) and were then given a recognition task, consisting of a list of 50 words formed by the words from List A, the words on List B and phonologically or semantically similar words. This test assesses immediate free recall, delayed free recall and recognition, in addition to measuring the effect of proactive and retroactive interference. It also provides a learning curve and qualitative information about the responses. This measure of verbal memory is highly dependent on both frontal and medial temporal lobes [54]. Several variables were recorded: number of words in the first trial in list A(AI); the total number of words from list A recalled across trials I to V (total A); the total number of words recalled from list B; the number of words in the immediate recall of list A (VI); the number of words recalled after a 20 minute delay (VII), intrusions (the total number of words that are not part of the list that is being considered at that moment), proactive interference (difference between the number of words recalled after the first presentation of list A and list B) and retroactive interference (difference between the number of words recalled in trial V and the number recalled in trial VI of list A) and recognition. We have used alternative versions of the task to reduce potential practice effects, despite the large interval between assessments.

Logical Memory subtest (Wechsler Memory Scale-III, WMS-III) [62]. In this task, two stories (A and B) were read to the participants, with story B being presented twice. After each story was read out, the subject was asked to reproduce it. After 30 minutes, the students were asked to say what they remembered of the story. Participants were then given a recognition test (15 true or false questions per story). Scores were recorded as follows: Logical Memory I (total immediate recall), measured as the sum of the score for recall of story A and the two presentations of story B, mainly reflecting codification; Logical Memory II (total delayed recall), measured as the sum of the score of delayed recall of story A plus delayed recall of story B, reflecting consolidation process; and recognition.

### Statistical Analysis

Generalized linear mixed models (GLMMs) with maximum log-likelihood approximated by adaptive Gauss-Hermite quadrature were applied to the data [63]. The models were implemented using the *lme4* package [64] in the R software environment [65]. All results were expressed as relative risks (RRs) and their 95% confidence intervals (CIs). Unlike classical analysis of repeated measures, GLMMs are particularly useful for longitudinal studies as they handle different number of participants at each evaluation. The models enable analysis of repeated measurements controlling correlated errors of measurement and individual heterogeneity, and they provide greater statistical power than ordinary regression models [66]. GLMMs can deal with non-normal data as in this case. The models were fitted with the negative binomial distribution for intrusions and perseverative errors in the RAVLT to obtain standard errors corrected for the overdispersion parameter (function: *glmer*.*nb*), and the Poisson family (*glmer*) was used for the other neuropsychological measures.

The scores of the neuropsychological tests were the dependent variables, with individual observations at each follow-up as level 1 and students (all individual observations at each follow-up nested in student) as level 2. Random effects among students were considered to control for intra-individual heterogeneity. The models were adjusted for time and sex. Several variables were tested to determine whether they had an explanatory role: frequency of cannabis use, frequency of tobacco use, age of drinking onset, and SCL-90-R GSI score. We first performed a bivariate analysis with these covariates. We then carried out a multivariate analysis in which we included all the independent variables with a statistical significance lower than 0.2. The nonsignificant independent variables were eliminated from this maximum model when the coefficients of the main exposure variables did not vary by more than 10% and the value of Schwartz's Bayesian Information Criterion (BIC) decreased. We corrected for multiple comparisons within each task using SidakSD (step-down adjusted p-values for strong control of the FWER [for positive orthant dependent test statistics]).

With the purpose of determining the progression of deficits, we used a separate GLMM for the different groups by comparing the performance in each follow-up relative to baseline. We also compared the neuropsychological performance of short-term ex-BDs and long-term ex-BDs relative to the stable non-BDs, to determine the effect of abandoning the BD pattern of alcohol consumption.

## Results

### Demographic and Substance Use Variables

The descriptive characteristics of the sample at baseline are shown in Table 1. Groups differed in age, *t*(152) = 2.86, *p* = .005. In particular, the BDs were slightly older than the non-BDs. Differences were found in the following variables: age of onset of alcohol use, *t*(137) = 4.83, *p* = .001; total AUDIT scores, *t*(124.32) = 15.68, *p* = .001; number of drinks per hour, *t*(153) = 14.48, *p* = .001; alcohol grams consumed during the week, *t*(73.61) = 8.44, *p* = .001, and alcohol grams consumed on the day of highest consumption, *t*(71.51) = 5.94, *p* = .001; and tobacco use, X^2^(2, N = 153) = 8.12, *p* = .004 and cannabis use, X^2^(2, N = 153) = 19.50, *p* = .001. None of the participants included in the study consumed cannabis daily. Eight participants were considered occasional users, as five of them consumed cannabis monthly and three of them consumed cannabis weekly. The other participants consumed cannabis less frequently than once a month or not at all. There was no differences in psychopathological symptoms measured by GSI scores of SCL-90-R test, *t*(153) = 0.76, *p* = .447. The groups did not differ in estimated intellectual level assessed by the Vocabulary subtest (WAIS-III) (Wechsler, 1997a). Those participants who completed the final follow-up were not statistically different from those who abandoned the study or were excluded throughout the study for any of the previous demographic and substance use variables. Table 2 depicts the demographic and substance use variables for each group over time.

**Table 1:** Group means (standard deviation) for demographic and clinical data at baseline.

	Non-BDs *n* = 76	BDs *n* = 79
Sex (m/f)	36/40	40/39
Age**	18.58 (0.60)	18.87 (0.63)
Age of onset alcohol use***	15.78 (1.04)	14.8 (1.30)
AUDIT total***	2.95 (2.58)	12.22 (4.55)
Number of drinks per hour***	1.04 (0.84)	3.39 (1.14)
Alcohol grams during the week ^a^***	42.19 (52.79)	312,41 (262.84)
Alcohol grams, day of highest consumption ^a^***	27.63 (31.92)	190.14 (234.71)
Occasional cannabis users^b^***	0	8
Tobacco smokers^b^**	3	24
GSI (SCL-90-R], Pc	46.39 (28.83)	50.09 (31.36)
WAIS-III Vocabulary	12.56 (1.97)	12.11 (1.62)

^*a*^ Week prior to the evaluation.

^b^ Monthly and weekly consumption. *Note*. Daily cannabis users were not included in the study.

BD, Binge Drinking.

**p < .01.

*** *p* < .001.

**Table 2:** Group means (standard deviation) for demographic and clinical data over time.

	First follow-up			Second follow-up			Third follow-up		
	Stable non-BD	Stable BD	Ex-BDs	Stable non-BD	Stable BD	Ex-BDs	Stable non-BD	Stable BD	Ex-BDs
Sex (m/f)	18/21	19/14	6/15	15/18	9/8	9/15	8/8	2/2	8/12
Age	20.71 (1.24)	21.07 (0.92)	21.08 (1.18)	22.81 (1.25)	22.97 (1.02)	23.23 (0.77)	25.49 (0.84)	25.40 (0.88)	24.97 (0.96)
Occasional cannabis use^b^	0	10	2	1	6	7	0	0	2
Tobacco smokers^b^	1	11	4	0	3	5	0	0	3
AUDIT total	2.92 (2.85)	11.73 (3.49)	6.43 (3.49)	2.64 (2.39)	11.13 (2.31)	7 (2.81)	1.80 (1.89)	10.33 (2.08)	6.58 (3.40)
Number of drinks per hour	0.82 (0.72)	2.55 (0.75)	1.13 (0.74)	0.68 (0.58)	2.31 (0.47)	1.15 (0.76)	0.60 (0.64)	2.67 (0.58)	1.11 (0.66)
Alcohol g during the week^a^	37.69 (33.96)	224 (132.74)	121.43 (80.77)	41.06 (41.02)	217 (118.46)	109.16 (67.98)	17.66 (29.99)	145 (32.78)	88.68 (55.19)
Alcohol g, day of highest consumption^a^	29.81 (24.72)	111.21 (48.29)	64.64 (31.65)	26.36 (23.82)	124.06 (57.64)	59.37 (28.80)	12.17 (18.44)	103.33(15.27)	55.53 (36.06)

^a^ Week prior to the evaluation.

^b^ Monthly and weekly consumption.

*Note*. Daily cannabis users were not included in the study.

g, grams.

### Binge Drinking Trajectory

The relationship between trajectory of consumption and episodic memory performance is summarised in Table 3. The analysis took into account the total number of data points throughout the study in each trajectory. The results of the RAVLT did not indicate any significant effect on word recall related to the BD trajectory over time (first trial list A, total A, total B, VI, VII) or in the recognition trials. No between-group differences were found in perseverative errors or susceptibility to proactive or retroactive interference. Stable BDs committed more intrusion errors than the non-BDs (Relative Risk [RR] = 1.65, 95% CI [1.02, 2.68], *p* = .041; SidakSD *p* = .342). In the Logical Memory subtest, a stable BD pattern was associated with poorer immediate recall (Logical Memory I), (RR = 0.94, 95% CI [0.89, 0.98], *p* = .011; SidakSD *p* = .022) and delayed recall of stories (Logical Memory II) (RR = 0.91, 95% CI [0.87, 0.97], *p* = .003; SidakSD *p* = .009) relative to stable non-BDs. The RR coefficient is the ratio of means of neuropsychological performance in stable BDs/stable non-BDs in the sample. The 95% confidence interval represents the range of values observed at a population level. This means that more difficulties in verbal memory were observed in delayed trials ([1/0.91] 10% risk) than in immediate trials ([1/0.94] 6% risk). The delayed recall of stories in stable BDs was 10% lower than in non-BDs, and it could be up to 15% lower (95% CI: 3%, 15%). The groups did not differ in their responses in the recognition trials.

**Table 3:** Generalized linear mixed models for neuropsychological scores and BD trajectory.

GLMM. Relative Risk (95% CI)		
	Stable BDs ^a^ (*n* = 133)	Ex-BDs ^a^ (*n* = 65)
**Logical Memory (WMS III)**		
Logical Memory I (total immediate recall)	0.94 [0.89, 0.98]*	0.95 [0.89, 1.00]
Logical Memory II (total delayed recall)	0.91 [0.87, 0.97]**	0.92 [0.86, 0.98]*
Recognition	0.99 [0.94, 1.05]	0.99 [0.93, 1.06]
**RAVLT**		
Trial I	0.99 [0.89, 1.10]	0.98 [0.87, 1.11]
Total List A	0.99 [0.94, 1.03]	0.99 [0.93, 1.04]
Total List B	0.98 [0.89, 1.09]	1.05 [0.93, 1.19]
Trial VI	0.96 [0.88, 1.04]	0.95 [0.86, 1.04]
Trial VII	0.98 [0.91, 1.06]	0.98 [0.89, 1.08]
Recognition List A	1.00 [0.89, 1.11]	1.00 [0.86, 1.15]
Proactive Interference	1.00 [0.97, 1.03]	1.00 [0.96, 1.03]
Retroactive Interference	1.00 [0.98, 1.03]	1.00 [.97, 1, 04]
Intrusion errors	1.65 [1.02, 2.68]	1.49 [0.86, 2.59]
Perseverative errors	1.29 [0.88, 1.89]	1.32 [0.87, 2.01]

CI, Confidence interval.

*Note*. This analysis examined episodic memory performance across 6 years on the basis of the BD trajectory.

^*a*^ Reference category: Stable non-BD trajectory (*n* = 164).

*p < .05.

**p < .01.

In relation to the effects of abandonment of the BD pattern of alcohol consumption, the ex-BDs did not differ significantly from the stable non-BDs in intrusion errors in the RAVLT (RR = 1.49, 95% CI [0.86, 2.59], *p* = .158; SidakSD *p* = .821). Furthermore, the ex-BDs did not differ from the stable non-BDs in immediate recall in the Logical memory test (Logical Memory I) (RR = 0.95, 95% CI [0.89, 1.00], *p* = .055; SidakSD *p* = .107), However, ex-BDs continued to show deficits (RR = 0.92, 95% CI [0.86, 0.98], *p* = .013; SidakSD *p* = .039) in delayed recall (Logical Memory II) (see Table 2). The ex-BDs performed similarly to stable BDs.

The sex x group interaction effect was not significant. We examined a possible dose-response effect by considering the speed of consumption (number of drinks per hour) and the effect of BD frequency (item 3 of the AUDIT). Neither of these variables were associated with poorer neuropsychological performance within the stable BDs. Age of drinking onset, frequency of cannabis use and frequency of tobacco use, psychopathological symptoms (GSI score of SCL-90-R) were controlled for, although none of the variables contributed significantly to explain the performance.

### Progression of Neuropsychological Performance

Stable BDs did not show any significant changes over time in the risk of committing intrusion errors in the RAVLT (Table 4). However, stable BDs committed fewer perseverative errors in the RAVLT at the first follow-up, relative to baseline (RR = 0.60, 95% CI [0.48, 0.76], *p*< .001). No other significant changes were observed for the other variables in this task in the BDs. In the Logical Memory subtest, there were no significant changes in immediate and delayed recall in stable BDs over time.

**Table 4:** Generalized linear mixed models for the progression in performance in stable BDs.

GLMM. Relative Risk (95% CI)			
	First follow-up ^*a*^	Second follow-up ^*a*^	Third follow-up ^*a*^
**Logical Memory (WMS III)**			
Logical Memory I (immediate recall)	1.02 [0.96, 1.08]	1.05 [0.96, 1.13]	1.03 [0.87, 1.23]
Logical Memory II (delayed recall)	1.04 [0.96, 1.13]	1.07 [0.96, 1.18]	1.10 [0.89, 1.36]
**RAVLT**			
Intrusion errors	1.20 [0.78, 1.86]	1.08 [0.58, 1.99]	0.43 [0.06, 3.29]

CI, Confidence Interval.

^*a*^ Reference category: baseline stable BDs.

The average performance of the stable non-BDs group did not differ significantly over time in the RAVLT. Stable non-BDs performed slightly better in delayed recall of stories at the second follow-up than at baseline (RR = 1.08, 95% CI [1.00, 1.18], *p* = .046). In this group, the performance in the Logical Memory did not present any significant variations throughout the study.

### Length of Time as Ex-Binge Drinkers

We grouped the ex-BDs into two categories to determine the effect of abandoning the BD pattern of alcohol consumption: short-term ex-BDs and long-term ex-BDs (Table 5). Neither short nor long-term ex-BDs differed significantly from the stable BDs regarding the risk of committing intrusion errors in the RAVLT. In immediate recall (Logical Memory I), short-term ex-BDs performed poorly (RR = 0.94, 95% CI [0.89, 0.99], *p* = .046) relative to the stable non-BDs, whereas long-term ex-BDs performed similarly to the stable non-BDs (RR = 0.96, 95% CI [0.91, 1.02], *p* = .200). Short-term ex-BDs exhibited difficulties in delayed recall (Logical Memory II), (RR = 0.91, 95% CI [0.86, 0.98], *p* = .008) while long-term ex-BDs did not show significant difficulties in the same variable (RR = 0.93, 95% CI [0.86, 1.00], *p* = .052), although the difference was almost statistically significant. There were no statistically significant differences between short-term and long-term ex-BDs or relative to BDs in any of the variables.

**Table 5:** Generalized linear mixed models for neuropsychological difficulties in participants who abandoned the BD pattern.

GLMM. Relative Risk (95% CI)		
	Short-term ex-BDs^a^ *n* = 33	Long-term ex-BDs^a^ *n* = 32
**Logical Memory (WMS III)**		
Logical Memory I (immediate recall)	0.94 [0.89, 0.99]*	0.96 [0.91, 1.02]
Logical Memory II (delayed recall)	0.91 [0.85, 0.98]**	0.93 [0.86, 1.00]
**RAVLT**		
Intrusion errors	1.56 [0.82, 2.99]	1.10 [0.51, 2.39]

Note. CI = Confidence Interval.

^a^ Reference category: stable non-BDs.

*p < .05.

**p < .01.

## Discussion

This study examined the relationship between episodic memory and BD trajectory during adolescence and early adulthood (age 18–23 years) in university students with no other risk factors. We first hypothesized that stable BDs would show executive deficits in a list-learning paradigm with non-semantically related words (RAVLT). Stable BDdid not exhibit difficulties in the RAVLT in learning, immediate or delayed recall of words or recognition. However, stable BDs tended to commit more intrusion errors, which may reflect impairment of self-monitoring functions [52, 54].

In a study using a long-term verbal learning task consisting of recalling words from a list of 36 non-semantically related words after a period of 25 minutes, Hartley et al., [67] did not observe any differences in delayed recall of words. As in the present study, BDs may have used alternative strategies to recall the list of non-semantically related words and thus compensate for possible limitations in episodic memory. In addition, qualitative information about types of errors or susceptibility to interference was not assessed, and the contribution of potential executive difficulties to episodic memory was not determined. In a recent study, Nguyen‐Louie and colleagues [68] showed that young BDs performed similarly to moderate drinkers in the CVLT, a task with semantically related words. Only extreme BDs (more than 10 drinks per occasion) had deficits in verbal learning and memory in this task [68]. Sneider et al., [34] found that young BDs demonstrated inferior learning (fewer words on the first and second learning trials) and recognition (omission and commission errors) in the CVLT, interpreted as verbal memory deficits. However, poor use of strategies related to a potential executive deficit may also lead to poor performance in list-learning tasks. Using another semantically related list-learning paradigm (Test de Aprendizaje Verbal España-Complutense [TAVEC]); García-Moreno and colleagues, [35, 36] observed more intrusion and perseverative errors in adolescent BDs, as well as poor performance in recognition tasks due to more false positive and omission errors. The authors attributed these differences to executive difficulties rather than to poor episodic memory. Our findings may be consistent with this idea. Similarly, Sanhueza et al., [37] showed that young BDs committed more perseverative errors in the same task (TAVEC), again indicating executive dysfunction.

We also expected that stable BDs would show difficulties in verbal episodic memory in a story-learning task, compatible with hippocampal dysfunction. In this regard, we found that a stable BD pattern of alcohol consumption is associated with difficulties in recalling stories in the Logical Memory subtest, which has been shown to depend on the left hippocampus [48]. Stable BDs exhibited deficits in both immediate (RR 6%) and delayed recall (RR 10%), probably reflecting encoding difficulties and particularly consolidation deficits in episodic memory. The groups performed similarly in recognition, which may be explained by the low difficulty of this trial (ceiling effect). No dose-response effect was found in the stable BDs, probably due to the low variability within this group. Finally, age of onset of alcohol consumption, psychopathological distress and frequency of cannabis use and tobacco did not contribute to explaining these results.

It is widely recognized that chronic alcohol abuse and that reduced hippocampal volume [69, 12] are associated with deficits in episodic memory [25]. Similarly, it has been found that young adults with AUD have a smaller hippocampal volume [26], especially the left hippocampus [27, 28]. In a study with university students, Howell and colleagues [70] reported significant correlations between hippocampus volume and AUDIT scores, although no structural abnormalities were found in this region. At the neuropsychological level, young adults with AUD had a 10% deficit in delayed recall of verbal information [29, 30]. To our knowledge, apart from the studies by our research group, no other studies have used a story-learning task to assess verbal episodic memory in young BDs. Ferret et al., [71] used the Children’s Memory Scale—a story-learning task- to evaluate South African adolescents with AUDs. The results showed that alcohol dependence was associated with poor performance in immediate and delayed recall of stories, as in the present study. The findings may be explained by the effects of alcohol on neural stem cell inhibition of adult neurogenesis in the adolescent hippocampus [72, 73]. During adolescence, the hippocampus displays greater neurogenesis than in adulthood. Consequently, excessive drinking during this period may result in a massive loss of cells that cannot be born or survive [73–75]. In adolescent rats, heavy drinking leads to long-term reduction of hippocampal neurogenesis, deficits in hippocampal-dependent memory [21–23] and inhibition of long-term potentiation (LTP) [76]. Accordingly, in adolescent non-human primates, Taffe and colleagues [77] found that BD was associated with a lasting reduction of hippocampal neurogenesis.

Regarding the progression of difficulties, we hypothesized two different trends suggesting different underlying mechanisms. On the one hand, previous studies with part of this sample [38, 39] showed that deficits in list B and greater proactive interference in the RALVT disappeared at the first follow-up, possibly suggesting delayed efficiency in executive functions in BDs relative to age-matched non-BDs. We thus hypothesized that executive deficits (e.g. intrusions) would improve over time, compatible with an alcohol-related neuromaturational lag. However, results showed that the number of intrusion errors did not change significantly over time. On the other hand, we found that poor recall of stories continued to occur at the first follow-up [39]. We therefore expected episodic memory deficits to remain stable or worsen, consistent with the special hippocampal vulnerability during adolescence [78]. In the present study, we found that the risk of poor episodic memory remained constant in stable BDs through adolescence to young adulthood. Likewise, in a 10-year follow up, the authors reported that clinical youths who chronically abused alcohol and other drugs had the poorest verbal memory in the CVLT over time, particularly delayed verbal recall and recognition [56].

Finally, improved performance was expected in young adults who abandoned the binge drinking pattern, indicating partial recovery. The results showed that relative to stable non-BDs, ex-BDs—both long and short term—did not display executive difficulties. In episodic memory, short-term ex-BDs still had difficulties in immediate (encoding) and delayed (consolidation) recall of stories, relative to stable non-BDs. However, long-term ex-BDs did not show any difficulties in episodic memory relative to stable BDs, although for consolidation the risk of presenting difficulties was almost significant (*p* = .052) in this group. Short-term and long-term ex-BDs performed similarly in immediate and delayed recall and no differences were observed in recalling of stories when both were compared with stable BDs. The findings indicate that consolidation deficits (which more entirely rely on the integrity of the medial temporal lobe) are particularly resistant to improvement, as long-term BDs displayed a position in performance between non-BDs and BDs but apparently closer to the last group. Overall, the results suggest that verbal episodic deficits continue to occur in the short-term and partial degree of recovery may be observed in the long term for encoding and to a less extent for consolidation of verbal information. Winward et al., [79] reported that after a four-week period of abstinence, adolescents partaking in heavy alcohol consumption showed some improvement in verbal memory. Despite the improvement, their performance was not as good as that of the young adults with a low level of alcohol consumption.

The main limitation of this study is the sample attrition. This mainly affects the last follow-up. Unlike classical repeated measures analysis, GLMMs are particularly useful in longitudinal designs, offering the advantage of being able to handle different number of participants in each evaluation. Therefore, the findings related to the trajectories of consumption (stable BDs or ex-BDs) are less affected by this limitation, as a greater number of data points are included. In addition, these models also consider the response correlation in repeated measures (such as correlated measurement errors and participant´s heterogeneity), resulting in greater statistical power [66]. One possible weakness of the study is the fact that while the AUDIT assesses alcohol use during the previous 12 months, information about alcohol consumption was collected every 22 months. There was therefore an undocumented period of 10 months. Nonetheless, we believe that the four evaluations carried out during 6 years provide an acceptable description of the different trajectories.

Another potential limitation of this study is the fact that we did not carry out any neuropsychological assessment before the initiation of BD, which may limit the interpretation of executive deficits, as explained before. However, the age of drinking onset, which constitutes a retrospective factor, did not play a role in explaining the neuropsychological performance in the present study. Regarding hippocampal-related difficulties, participants who abandon the BD seem to show some degree of recovery in the long term, possibly suggesting that the difficulties are a consequence of BD rather a pre-existing feature. In line with this interpretation, a neuroimaging study found that hippocampal volume before the start of alcohol consumption in adolescents (12–14 years old) did not predict AUD four years later [80]. These findings indicate that differences in hippocampal volume are related to the neurotoxic effects of alcohol and do not precede heavy drinking.

We report the finding of intrusion errors with caution. We have followed a conservative approach and corrected for multiple comparisons (SidakSD), which resulted in a nonsignificant p-value for this variable. Nonetheless, a greater number of intrusion errors in a healthy population with no other risk factors is of clinical relevance and is also consistent with previous findings. Moreover, correcting variables that may be related to different processes (such as “total list A” and “intrusions”) may lead to an overcorrection (Type II error). As a final consideration, no alternative versions were used in the Logical Memory test. Even though there were large intervals between assessments, some minimal practice effects may have influenced the absence of any changes in performance (no further worsening) over time in BDs.

In summary, maintaining a BD pattern of alcohol consumption from adolescence through to early adulthood is associated with episodic deficits—especially in memory consolidation—together with possible executive difficulties that also contribute to poor verbal memory performance in healthy university students. Memory plays a crucial role in life, but is particularly important during college years when difficulties in memorizing verbal information may have a great impact on academic life. Abandoning the BD pattern may lead to partial recovery. Nevertheless, there is a pressing need to integrate the scientific evidence to strengthen prevention programmes that raise awareness of the cognitive impacts of BD, principally among university students.
